# Dietary supplementation with honeycomb extracts positively improved egg nutritional and flavor quality, serum antioxidant and immune functions of laying ducks

**DOI:** 10.3389/fvets.2023.1277293

**Published:** 2023-10-10

**Authors:** Xiaolian Chen, Pingwen Xiong, Wenjing Song, Qiongli Song, Zhiheng Zou, Jiangnan Huang, Jiang Chen, Chuanhui Xu, Weide Su, Gaoxiang Ai, Qipeng Wei

**Affiliations:** Institute of Animal Husbandry and Veterinary Science, Jiangxi Academy of Agricultural Sciences, Nanchang, China

**Keywords:** honeycomb extracts, laying performance, nutritional and flavor quality, antioxidant function, immune function, laying duck

## Abstract

**Introduction:**

Honeycomb is a traditional natural health medicine and has antioxidant, antibacterial, anti-inflammatory, antiviral and antitumor activities. It is currently unclear whether honeycomb extract supplementation has positive effects on the intensive farming laying duck production. This study aims to evaluate the effects of honeycomb extracts on the laying performance, egg nutritional and flavor quality, serum biochemical indexes, and antioxidant and immune status in laying ducks.

**Methods:**

A total of 672 healthy 28-week-old Shanma laying ducks with similar laying performance and body weight were randomly distributed into four dietary treatments with 6 replicates of 28 birds. The birds in each treatment were fed the basal diet supplemented with 0 (control group), 0.5, 1.0 or 1.5 g/kg honeycomb extracts, respectively. Feed and water were provided *ad libitum* for 45 days. Laying performance, egg quality, egg nutrition and flavor quality, serum parameters were assessed.

**Results:**

The results showed that compared with the control group, honeycomb extracts addition significantly increased the average daily feed intake but did not affect the other laying performance indexes, egg quality or serum biochemical indexes of laying ducks. Dietary supplementation with honeycomb extracts significantly increased crude protein content and decreased the contents of cholesterol and trimethylamine in eggs. Diets supplemented with 1.5 g/kg honeycomb extracts significantly improved egg total amino acids and flavor amino acids contents, monounsaturated fatty acids and polyunsaturated fatty acids composition and enhanced the serum antioxidant activity and immune functions of ducks.

**Discussion:**

Duck eggs are rich in nutrients and a valuable source of high-quality food for human, while they are rarely consumed directly by consumers because of their stronger fishy odor and lower sensory quality. Many studies have showed that the influence of dietary supplementation on egg components. This study indicated that dietary supplementation with honeycomb extracts positively reduced the contents of egg cholesterol and trimethylamine, improve egg amino acids contents and fatty acid profiles, enhanced serum antioxidant and immune status of laying ducks. The recommended supplemental level of honeycomb extracts was 1.5 g/kg in the diet of laying ducks.

## 1. Introduction

Poultry eggs are one of the most common palatable and nutritional food. Duck eggs are rich in protein and amino acids, fatty acids, minerals, and vitamins, which provide a valuable source of high-quality nutrients for human food and health. However, fresh duck eggs are rarely consumed directly by consumers because of their stronger fishy odor and lower sensory quality, which closely related to the egg nutrition components. With the rapid development of economy and the continuous improvement of people’s living standards, people put forward higher quality requirements for nutritional and health quality of eggs. Therefore, it is urgent to improve the nutrition and health quality of duck eggs.

Egg amino acids are the most ideal source of high-quality protein in natural foods and are easily absorbed and utilized. At the same time, amino acids contribute notably to taste and flavor of eggs. Generally, the types of amino acids can be divided into several categories according to their taste characteristics, and flavor amino acids mainly include aspartic acid, glutamic acid, glycine acid, alanine acid, tyrosine acid and phenylalanine acid (1, 2). Egg fatty acids, especially polyunsaturated fatty acids (PUFAs), are important in poultry production for improving the health and productivity of birds, and are useful for improving cardiovascular health and cognitive function of human. Many studies have showed that the influence of dietary supplementation of vegetable seeds and oils, fish oil and natural antioxidants on the fatty acids composition of eggs (3–6). Another way to improve the health quality of eggs is through reducing the content of cholesterol and trimethylamine (TMA). Cholesterol plays a crucial role in the regulation of embryonic development and membranes function, but there is a growing consensus that elevated levels of blood cholesterol is associated with an increased incidence of cardiovascular disease in human (7). Consumers regard eggs as a high-cholesterol dietary product, marketing trends in the egg industry begin to turn their attention toward improving the health quality of eggs through reducing the cholesterol content in eggs. Previous studies have confirmed TMA is a major component that causes fishy odor of duck eggs (8). TMA production is closely associated with genetics, diet and gut microbiota (9, 10). Tian et al. (1) found that microbial fermented feed could reduce the TMA content of duck eggs.

In addition, laying ducks are very susceptible to the adverse consequences of stress in the commercial large-scale intensive production (11), which further reduce feed efficiency and cause immunosuppression and nutrient malabsorption (12). The decreased antioxidant and immune capacity subsequently decreased the potential production capacity. A growing evidence indicates that most of stresses in caged laying ducks production are associated with oxidative stress due to excess of free radical production or inadequate antioxidant protection (13). There has been a tendency towards using antioxidant supplementation, especially from natural sources to maintain high productive and reproductive performances and health of commercial intensive farming birds.

A large number of evidences indicated that duck production performance and egg components could be influenced by the diets of birds (14, 15). Honeycomb is a bee byproduct and a natural health care medicine and rich in flavonoids, polyphenols and polysaccharides (16–18), which have been shown to have antioxidant, antibacterial, anti-inflammatory, antiviral, and antitumor activities (19). Traditional and folk medicines were long ago shown to have many beneficial attributes as they could use phenol groups to scavenge free radicals and relieve the oxidative stress common to many diseases (20). Xu et al. (21) reported that wasp honeycomb extract can reduce free radical oxidation and infiltration in gastric tissue and protect rats from gastric injury caused by acidified ethanol. Many previous research results have showed that bee byproducts, such as honey (22), propolis (23, 24), and pollen (25), in the animal diet has a positive effect on performance and health.

Because of its beneficial effects on animal production, antioxidant activity and other physiological mechanisms (26), honeycomb has attracted much attention in the poultry industry. However, very limited information on honeycomb extracts in animal husbandry is available. We observed that honeycomb extracts could improve growth performance, carcass traits, immune function, serum antioxidant capacity and intestinal microorganisms in broilers (27). It is crucial to increase laying performance, promote immunity, suppress inflammation, and even improve egg nutritional quality in laying fowl. It is currently unclear whether honeycomb extract supplementation has positive effects on the intensive farming laying duck production. The present study aims to evaluate the effects of dietary supplementation with honeycomb extracts on the laying performance, nutrition and flavor quality, serum biochemical indexes, immune and antioxidant status of laying ducks. The research results will provide a theoretical basis and reference for further utilization of honeycomb to improve food safety and quality.

## 2. Materials and methods

### 2.1. Sample preparation

The honeycombs from a bee farm were dried, ground and extracted by ultrasound-assisted ethanol extraction using 60% ethanol at 50°C for 30 min with a solid–liquid ratio of 1:30 (g/mL). The liquid honeycomb extracts were concentrated and freeze-dried to obtain honeycomb extracts powder. The honeycomb extracts contain 12.68 mg/g of total flavonoids, 5.3 mg/g of polysaccharides and 108.8 mg/g of polyphenols.

### 2.2. Ducks, diets, and management

A total of 672 healthy 28-week-old Shanma laying ducks with similar laying performance and body weight (1.24 ± 0.02 kg) were chosen. After a one-week adaptation period, all ducks were randomly distributed into 4 dietary treatments with 6 replicates in each treatment group and 28 ducks per replicate. The basal diet without honeycomb extracts was used as a control group, and three increasing concentrations of honeycomb extracts, 0.5, 1.0 and 1.5 g/kg, were added to basal diet for the experimental groups. The experimental honeycomb extracts and premix are pre-mixed well into a homogeneous mixture. The basal diet was formulated to meet the nutrient requirements of egg duck (GB/T 41189-2021, China). The ingredients and chemical composition of the basal diet is presented in Table 1. The birds were housed in three-layer galvanized separate cages (20 cm wide × 38 cm deep × 38 cm tall, one bird per cage) and were allowed *ad libitum* access to feed and water. The birds were vaccinated and managed according to the breed standards. The light program consisted of 16:8 light: darkness cycle through a 45-d experimental period.

**Table 1:** Ingredients and chemical composition of the basal diet (air dry basis).

Items	Content
Ingredients, %	
Corn	46.50
Soybean meal	28.00
Rice bran	6.00
Wheat middling	4.00
Puffed soybeans	3.00
Rapeseed meal	2.00
CaHPO_4_	0.80
Limestone	8.40
NaCl	0.30
Permix^a^	1.00
Total	100.0
Chemical composition^b^	
Metabolizable energy (MJ/kg)	10.43
Crude protein, %	17.00
Calcium, %	3.60
Available phosphorus, %	0.35
Total phosphorus, %	0.65
Lysine, %	0.87
Methionine, %	0.40

a Permix provided the following per kilogram of diet: Vitamin A 8000 IU, Vitamin D_3_ 1000 IU, Vitamin E 20 mg, Vitamin K_3_ 2.0 mg, Vitamin B_1_ 2 mg, Vitamin B_2_ 15 mg, Vitamin B_6_ 6 mg, Vitamin B_12_ 0.02 mg, D-pantothenic acid 20 mg, nicotinic acid 60 mg, choline chloride 1000 mg, biotin 0.2 mg, folic acid 0.6 mg, antioxidant 100 mg, Fe 80 mg, Cu 10 mg, Mn 100 mg, Zn 60 mg, I 0.5 mg, Se 0.4 mg.

b Calculated values. Estimated from the Chinese feed database, which provides tables of feed composition and nutritive values in China (2021 32nd edition).

### 2.3. Laying performance

Egg production and egg weight were recorded daily, and feed consumption was recorded weekly per replicate. The average daily feed intake, laying rate, average egg weight, average daily egg yield, feed-to-egg ratio and qualified egg rate were calculated. The laying rate was calculated as the number of eggs produced/number of laying ducks × 100. The feed-to-egg ratio was calculated as total feed consumption/total egg weight × 100. The qualified egg rate was calculated as the number of total qualified eggs excluding defects/number of total eggs × 100.

### 2.4. Egg quality

Four eggs per replicate (a total of 24 eggs from each treatment) were collected and analyzed on day 45 for egg physical quality. Eggshell strength was measured on the vertical axis by a compression tester (EFG-0503, Robotmation, Tokyo, Japan). Eggshell thickness was measured by a micrometer and calculated as the average value of measurements at three points on the eggs (blunt end, equator, and sharp end). The egg shape index was determined by a caliper with the least count of 0.01 mm and was represented by the ratio of egg length to width. Egg yolk color, Haugh unit and albumen height were analyzed by an Egg Multi-tester (EMT-5200, Robotmation Tokyo, Japan). The yolk and albumen were isolated and weighed to calculate their percentages of egg weight. The measurements of the above indicators were completed within 48 h after laying.

### 2.5. Egg nutrition and flavor quality

Four eggs per replicate were chosen for chemical analysis on day 45. The crude protein and moisture of egg were measured according to AOAC methods (28). An improved method for cholesterol determination was used by HPLC as previously described (29). The TMA contents were determined with the colorimetric method described by Li et al. (9). The amino acid content and fatty acid profiles of the eggs were analyzed as described by Cullere et al. (30).

### 2.6. Serum biochemical parameters, antioxidant, and immune status

At 45 days of the experimental period, after 8 h of starvation, blood samples were taken from the wing vein from 2 ducks per replicate of each group. Standing at room temperature for one hour, serum was separated by centrifugation at 3,000 × g for 15 min and stored at −20°C for serum biochemical parameter analysis. Serum concentrations of albumin (23), total protein (TP), total cholesterol (31), triglyceride (TG), high-density lipoprotein (HDL), low-density lipoprotein (LDL), and glucose (32), the activities of superoxide dismutase (SOD) and glutathione peroxidase (GSH-Px), and content of malondialdehyde (MDA) were determined with commercial kits (Jiancheng Bioengineering Institute, Nanjing, China). The immune function indexes, including immunoglobulin A (IgA), IgG, IgM, interleukin-1β (IL-1β), IL-6 and tumor necrosis factor-α (TNF-α), were determined using commercial ELISA kits (Beijing Sino-UK Institute of Biological Technology, Beijing, China).

### 2.7. Statistical analysis

Statistical analyses were performed using SPSS version 20.0 for Windows (SPSS Inc., Chicago, IL, United States). The significance of mean differences between groups was made by one-way ANOVA followed by Tukey’s multiple range test. A dietary treatment replicate was used as the experimental unit for laying performance, and an egg or a duck in each replicate was selected as the experimental unit for egg physicochemical traits or serum parameters, respectively. The results are presented as the means with standard errors (SEM). Statistical differences were considered significant at 5% level (*p <* 0.05).

## 3. Results

### 3.1. Laying performance

The effects of dietary honeycomb extract supplementation on the laying performance of laying ducks are shown in Table 2. Compared with the control group, the laying ducks fed diets containing 0.5 and 1.0 g/kg honeycomb extracts showed significantly increased average daily feed intake (*p* < 0.05), while the average egg weight, average daily egg weight, feed to egg ratio and qualified egg rate were not affected by the dietary treatments (*p* > 0.05).

**Table 2:** Effects of dietary honeycomb extracts on the laying performance of laying ducks.

Items	Honeycomb extract level, g/kg				SEM	*p* value
	Control	0.5	1.0	1.5		
Average daily feed intake, g/d	163.29^b^	170.12^a^	169.30^a^	167.06^ab^	1.08	0.010
Laying rate, %	90.42	91.64	92.08	92.78	0.69	0.712
Average egg weight, g	68.68	69.28	69.13	68.67	0.19	0.610
Average daily egg yield, g	62.09	63.48	63.65	63.63	0.47	0.606
Feed to egg ratio	2.63	2.68	2.61	2.59	0.02	0.431
Qualified egg rate, %	99.41	99.65	99.58	99.70	0.10	0.789

SEM, standard error of the mean (*n* = 24). Data were analyzed by one-way ANOVA. ^a, b^Means within a row without a common superscript differ (*p* < 0.05).

### 3.2. Egg quality

The effects of supplementation with different level honeycomb extracts on the egg physical quality of laying ducks are shown in Table 3. No significant differences were observed in yolk color, albumen height, Haugh unit, egg shape index, eggshell strength and thickness, or percentage of yolk and albumen among groups (*p* > 0.05).

**Table 3:** Effects of dietary honeycomb extracts on the physical quality of eggs from laying ducks.

Items	Honeycomb extract level, g/kg				SEM	*p-*value
	Control	0.5	1.0	1.5		
Yolk color	10.25	10.42	10.43	10.43	0.07	0.781
Albumen height, mm	6.80	7.11	7.03	7.24	0.09	0.352
Haugh unit	77.93	79.72	79.33	80.96	0.60	0.377
Egg shape index	1.33	1.33	1.33	1.33	0.01	0.958
Eggshell strength, kg/cm^2^	4.65	4.87	4.79	4.88	0.07	0.633
Eggshell thickness, mm	0.47	0.46	0.47	0.48	0.00	0.461
Yolk ratio, %	33.35	34.00	33.70	34.19	0.30	0.779
Albumen ratio, %	53.20	52.11	52.06	51.65	0.34	0.406

SEM, standard error of the mean; *n* = 96 (4 samples per replicate).

### 3.3. Egg nutrition and flavor quality

Table 4 shows the effect of dietary honeycomb extracts on egg proximate composition and contents of cholesterol and TMA. The moisture content of eggs from ducks receiving a diet containing 0.5 g/kg honeycomb extract supplementation was significantly lower than that of eggs from ducks in the control group (*p <* 0.05). The crude protein content of eggs increased with the level of honeycomb extract added and was significantly different from the control when the level of honeycomb extract was 1.0 and 1.5 g/kg. Cholesterol and TMA were linearly decreased by supplementation with honeycomb extracts; these indexes were significantly lower in all experimental treatments than in the control (*p <* 0.05).

**Table 4:** Effects of honeycomb extracts on the proximate composition and cholesterol and trimethylamine contents of duck eggs.

Items	Honeycomb extract level, g/kg				SEM	*p*-value
	Control	0.5	1.0	1.5		
Moisture, %	70.72^a^	70.39^b^	70.47^ab^	70.42^ab^	0.056	0.001
Crude protein, %	12.39^c^	12.54^bc^	12.64^ab^	12.77^a^	0.036	0.043
Cholesterol, mg/g	6.10^a^	5.94^b^	5.91^b^	5.90^b^	0.021	0.002
Trimethylamine, μg/g	0.15^a^	0.14^b^	0.13^b^	0.13^b^	0.001	0.003

SEM, standard error of the mean; *n* = 48 (2 samples per replicate). ^a,b,c^Means within a row without a common superscript differ (*p* < 0.05).

The effects of honeycomb extracts on the amino acid profile of duck eggs are presented in Table 5. Dietary supplementation with honeycomb extracts significantly increased the contents of total amino acids, flavor amino acids, cystine, valine and histidine (*p <* 0.05). There were no differences among the groups in the threonine, glycine and lysine contents of eggs (*p >* 0.05). Dietary supplementation with 1.0 and 1.5 g/kg honeycomb extracts significantly increased the contents of alanine, isoleucine, leucine and tyrosine in the eggs of ducks (*p <* 0.05). The content of proline was significantly lower in the eggs of ducks receiving the diet containing 0.5 g/kg honeycomb extracts than in those from ducks receiving the control diet (*p <* 0.05).

**Table 5:** Effects of honeycomb extracts on the amino acid profile of duck eggs (%).

Items	Honeycomb extract level (g/kg)				SEM	*p*-value
	Control	0.5	1.0	1.5		
Asparagine	1.022^a^	1.019^ab^	0.992^b^	1.027^a^	0.005	0.042
Threonine	0.693	0.698	0.697	0.692	0.002	0.471
Serine	0.943^b^	0.954^ab^	0.962^a^	0.966^a^	0.002	0.001
Glutamic acid	1.678^b^	1.715^ab^	1.748^a^	1.755^a^	0.007	<0.001
Proline	0.479^a^	0.464^b^	0.476^a^	0.476^a^	0.001	<0.001
Glycine	0.407	0.403	0.407	0.410	0.001	0.105
Alanine	0.565^b^	0.576^ab^	0.589^a^	0.583^a^	0.003	0.003
Cystine	0.243^c^	0.255^b^	0.265^a^	0.266^a^	0.002	<0.001
Valine	0.615^b^	0.666^a^	0.675^a^	0.680^a^	0.005	<0.001
Methionine	0.744^b^	0.733^b^	0.748^a^	0.753^a^	0.002	<0.001
Isoleucine	0.519^b^	0.519^b^	0.538^a^	0.537^a^	0.002	<0.001
Leucine	0.966^b^	0.993^b^	1.045^a^	1.047^a^	0.007	<0.001
Tyrosine	0.531^b^	0.532^b^	0.547^a^	0.548^a^	0.002	<0.001
Phenylalanine	0.723^b^	0.737^ab^	0.752^a^	0.752^a^	0.003	<0.001
Lysine	0.912	0.925	0.921	0.938	0.006	0.476
Histidine	0.263^b^	0.275^a^	0.282^a^	0.281^a^	0.002	<0.001
Arginine	0.723^a^	0.715^ab^	0.712^b^	0.721^ab^	0.001	0.016
Total amino acids	12.020^c^	12.178^b^	12.355^a^	12.430^a^	0.026	<0.001
Flavor amino acids^1)^	4.926^c^	4.984^bc^	5.042^ab^	5.070^a^	0.013	<0.001

SEM, standard error of the mean; *n* = 48 (2 samples per replicate). ^a, b, c, d^Means within a row without a common superscript differ (*p* < 0.05). ^1)^Flavor amino acids include aspartic acid, glutamic acid, glycine acid, alanine acid, tyrosine acid and phenylalanine acid.

As shown in Table 6, compared with the control diet, supplementation with honeycomb extracts led to a decrease in the proportion of total saturated fatty acids (SFAs) and C10:0, C14:0, C16:0 and C20:0 in eggs (*p <* 0.05). Supplementation with honeycomb extract significantly increased the total unsaturated fatty acids (UFAs), total monounsaturated fatty acid (MUFA), total polyunsaturated fatty acids (PUFAs) and C18:1, C18:3n3 and C22:2 contents in eggs (*p <* 0.05).

**Table 6:** Effects of honeycomb extracts on the fatty acid of duck eggs (%).

Items	Honeycomb extract level, g/kg				SEM	*p*-value
	Control	0.5	1.0	1.5		
Capric acid (C10:0)	1.310^a^	1.201^b^	1.161^b^	1.122^b^	0.016	<0.001
Lauric acid (C12:0)	0.032^a^	0.030^ab^	0.029^b^	0.030^ab^	0.000	0.029
Myristic acid (C14:0)	0.322^a^	0.310^b^	0.294^c^	0.302^bc^	0.003	<0.001
Palmitic acid (C16:0)	19.161^a^	18.488^b^	18.904^ab^	18.520^b^	0.089	0.014
Margaric acid (C17:0)	0.091^a^	0.089^ab^	0.087^b^	0.087^b^	0.001	0.007
Stearic acid (C18:0)	4.238^a^	4.209^ab^	4.165^ab^	4.103^b^	0.017	0.024
Arachidic acid (C20:0)	0.015^a^	0.013^bc^	0.014^ab^	0.012^c^	0.000	0.001
Tricosylic acid (C23:0)	0.071^a^	0.068^b^	0.069^ab^	0.069^ab^	0.000	0.049
Saturated fatty acids	25.323^a^	24.487^b^	24.800^b^	24.324^b^	0.087	<0.001
Myristic acid (C14:1)	0.038	0.040	0.039	0.037	0.000	0.065
Palmitic acid (C16:1)	2.053^bc^	2.120^ab^	2.134^a^	2.042^c^	0.011	0.002
Oleic acid (C18:1)	50.024^b^	50.505^a^	50.577^a^	50.672^a^	0.062	<0.001
Oleic acid (C18:2)	7.479	7.630	7.514	7.586	0.028	0.208
Oleic acid (C18:3, n3)	0.094^c^	0.097^b^	0.105^a^	0.098^b^	0.000	<0.001
Oleic acid (C18:3, n6)	0.016^b^	0.016^b^	0.021^a^	0.018^ab^	0.001	0.012
Arachidic acid (C20:1)	0.362^b^	0.379^a^	0.357^bc^	0.350^c^	0.002	<0.001
Arachidic acid (C20:2)	0.052	0.049	0.052	0.050	0.000	0.071
Arachidic acid (C20:3)	0.122	0.120	0.124	0.124	0.001	0.220
Arachidic acid (C20:4)	1.850^ab^	1.843^ab^	1.810^b^	1.854^a^	0.006	0.022
Arachidic acid (C20:5)	0.089	0.088	0.090	0.090	0.001	0.677
Docosadienoic acid (C22:2)	12.051^c^	12.292^a^	12.071^b^	12.444^a^	0.364	<0.001
Docosatetraenoic acid (C22:4)	0.253	0.108	0.111	0.110	0.026	0.117
Docosahexaenoic acid (C22:6)	0.195^b^	0.198^ab^	0.197^ab^	0.201^a^	0.001	0.049
Monounsaturated fatty acids	52.477^b^	53.073^a^	53.109^a^	53.101^a^	0.063	<0.001
Polyunsaturated fatty acids	22.200^b^	22.443^a^	22.093^b^	22.576^a^	0.056	0.006
Unsaturated fatty acids	74.677^c^	75.513^a^	75.200^b^	75.676^a^	0.087	<0.001
n-3 Polyunsaturated fatty acids	0.378^b^	0.383^ab^	0.392^a^	0.389^a^	0.002	0.004
n-6 Polyunsaturated fatty acids	9.345	9.489	9.344	9.458	0.028	0.138
n-6 /n-3	24.742^a^	24.806^a^	23.883^b^	24.342^ab^	0.108	0.005

SEM, standard error of the mean; *n* = 48 (2 samples per replicate). ^a, b, c^Means within a row without a common superscript differ (*p* < 0.05).

### 3.4. Serum biochemical parameters, antioxidant, and immune status

As presented in Table 7, dietary supplementation with honeycomb extracts did not affect serum biochemical parameters (*p >* 0.05). The activity of serum SOD was higher in the laying ducks fed the honeycomb extract diet than in those in the control group (*p <* 0.05). There were no significant differences in the GSH-Px activity or MDA content of ducks receiving the honeycomb extract diet (*p >* 0.05). The concentrations of IgA, IgG and IgM were significantly higher in ducks fed a 1.5 g/kg honeycomb extract diet than in those in the control group (*p <* 0.05). The levels of serum IL-1β, IL-6 and TNF-α showed a significant linear response (*p <* 0.05) to supplementation with honeycomb extracts; overall, these levels were significantly lower in ducks consuming the 0.5–1.5 g/kg honeycomb extract diets than in those in the control group (*p <* 0.05).

**Table 7:** Effects of honeycomb extracts on serum biochemical parameters and antioxidant and immune indexes of laying ducks.

Items	Honeycomb extract level, g/kg				SEM	*p*-value
	Control	0.5	1.0	1.5		
Albumin, g/L	14.68	15.16	15.52	15.84	0.29	0.538
Total protein, g/L	59.84	60.43	60.92	61.87	1.06	0.924
Total cholesterol, mmol/L	3.50	3.14	3.05	2.94	0.14	0.562
Triglyceride, mmol/L	9.59	9.49	8.99	8.95	0.64	0.979
High-density lipoprotein, mmol/L	0.78	0.73	0.60	0.78	0.03	0.224
Low-density lipoprotein, mmol/L	0.58	0.73	0.64	0.64	0.04	0.630
Glucose, mmol/L	10.53	11.24	11.23	10.65	0.15	0.785
Glutathione peroxidase, U/mL	802.55	884.33	920.31	815.85	23.99	0.255
Malondialdehyde, nmol/mL	4.98	4.77	4.60	4.79	0.16	0.887
Superoxide dismutase, U/mL	68.02^b^	86.48^a^	91.99^a^	84.76^a^	2.43	0.002
IgA, g/L	1.35^b^	1.39^ab^	1.42^ab^	1.46^a^	0.01	0.047
IgG, g/L	2.49^b^	2.65^ab^	2.69^ab^	2.78^a^	0.04	0.014
IgM, g/L	0.97^b^	1.08^ab^	1.10^ab^	1.12^a^	0.02	0.049
Interleukin-1β, pg/mL	17.37^a^	16.39^ab^	14.58^bc^	13.10^c^	0.44	0.001
Interleukin-6, pg/mL	189.66^a^	163.85^b^	148.69^b^	122.14^c^	4.54	<0.001
Tumor necrosis factor-α, pg/mL	67.29^a^	57.10^b^	53.14^bc^	47.24^c^	1.63	<0.001

SEM, standard error of the mean; *n* = 48 (2 samples per replicate). ^a, b, c^ Means within a row without a common superscript differ (*p* < 0.05).

## 4. Discussion

In recent years, green and high-efficiency traditional Chinese medicine extracts feed additives as natural growth promoters have attracted broad interest in the poultry industry. The present study was designed to investigate the effects of dietary honeycomb extracts on the laying performance, egg nutrition and flavor quality and blood parameters of laying ducks. The results showed that dietary supplementation with honeycomb extracts had no significant effects on the average egg weight, average daily egg weight, feed to egg ratio and qualified egg rate of laying ducks but increased the average daily feed intake of laying ducks and did not affect egg physical quality, such as yolk color, Haugh unit, and shell thickness. In general, Chinese medicine extracts, especially those rich in flavonoids and phenolic acids, are well-known to improve the health and egg production of poultry (33, 34). Zhou et al. (35) showed that dietary flavonoid supplementation with 3 mg/kg increased the laying rate, average egg weight and the feed conversion ratio. Other studies reported that supplementation with flavonoids and phenolic acids had no effect on egg production and egg weight (36, 37). Iskender et al. (32) reported that dietary flavonoids and phenolic acids have no effect on poultry production in either laying performance or eggshell quality. These inconsistent results might be due to different types of extracts, poultry and diets. Because of the limited studies on the effect of honeycomb extracts on animals, further study is still necessary.

Duck eggs are rich in nutrients and contain all essential amino acids required in the human diet. Although duck eggs are more nutrient dense than chicken eggs, the higher cholesterol content and fishy odor limit their consumption as table eggs (9, 38). The chemical composition of eggs can be modified through dietary manipulation. As an example, omega-3 fatty acid levels in eggs can be elevated by feeding diets rich in omega-3 fatty acid sources, such as canola oil, soybean oil, flaxseed and walnut (14, 39). In the present study, compared with the control group, dietary supplementation with honeycomb extracts significantly increased the egg crude protein content and significantly decreased the moisture, cholesterol and TMA contents. Cholesterol is an important metabolite of cell membrane structure and myelin, a precursor of steroid hormones and play a vital role in biological functions (40). However, changes in plasma cholesterol levels have been found to be directly related to cardiovascular disease events in young adults (41). Therefore, decreasing the cholesterol concentration of eggs can improve egg nutritional quality and market competitiveness, which is beneficial to human health. The cholesterol-decreasing effect of flavonoids may be linked to the inhibition of hydroxy-methyl glutaryl coenzyme A reductase activity, the first step enzyme in cholesterol synthesis (42–44), the biological effect could be related to the chemical structure of flavonoids (45).

The TMA in eggs usually comes from dietary sources, TMA precursors, such as lecithin, betaine and carnitine, and choline-rich feed (31). Dietary choline and TMA precursors can be metabolized by intestinal microorganisms to form TMA (46). In the present study, compared with the control diet, supplementation with honeycomb extracts significantly decreased the TMA concentration. The decrease in TMA might be because supplementation with honeycomb extracts inhibited related microorganisms from cleaving choline into TMA; however, the exact reasons and mechanisms need to be further investigated.

Total UFAs, MUFAs and PUFAs increased with increasing dietary honeycomb extract levels in the current study. As shown in Table 6, the dietary honeycomb extracts significantly decreased the contents of the total SFAs in the eggs of ducks. In previous studies, both *in vitro* and *in vivo* studies suggested that SFAs can activate pro-inflammatory signaling pathways, leading to insulin resistance (47). Other studies also demonstrated that SFAs indeed can activate Toll-like receptor 4-(TLR4-) and TLR2-mediated pro-inflammatory signaling pathways and consequently increase the risk of insulin resistance (48). In addition, previous studies indicated that PUFAs such as docosahexaenoic acid (C22:6) and arachidonic acid (C20:4) play vital roles in brain development during infancy (49), and adenoma cells are highly susceptible to PUFA-induced apoptosis (50). Some studies also showed that replacing saturated fat with monounsaturated fat is efficacious at reducing the total high-density lipoprotein cholesterol ratio (51). In the present study, the contents of C16:1, C18:1, C18:3n3, C22:2 and total UFAs, MUFAs and PUFAs in eggs of ducks were significantly increased. Which can prevent unsaturated fatty acids on the cell membrane from being oxidized.

Moreover, dietary supplementation with honeycomb extracts significantly increased the contents of total amino acids and flavor amino acids. The results verified that honeycomb extract could improve the nutritional value of duck eggs. This possibly due to honeycomb extracts can increase antioxidant potential and might take part in the amino acids metabolic signaling pathways and regulate gene expression (52). However, the exact molecular mechanism of this effect remains obscure. This suggests that further work is still needed to evaluate the specific mechanism of honeycomb extracts to ascertain their efficacy. Our observations were consistent with Li et al. (53), who found that feeding of anthocyanins from purple corn extract could improve flavor amino acids content in eggs compared to the control group.

Honeycomb, as a natural product, has many beneficial attributes, including antioxidative, anti-inflammatory, and antibacterial properties. Khodabakhshi et al. (54) reported that the antimicrobial activity of honeycomb extract is attributed to the presence of bioactive compounds such as flavonoids, which show antibacterial and anti-inflammatory effects and might improve the health of animals. Other studies also reported that honeycomb extract application may serve as a method for treating inflammatory diseases by reducing inflammation and free oxygen radical production (19). In the present study, blood analysis suggested that the levels of IgA, IgG and IgM in the honeycomb extract treatment group were significantly higher than those in the control group, and the levels of serum IL-1β, IL-6, and TNF-α were significantly lower in ducks consuming honeycomb extract diets than in those in the control group, suggesting that honeycomb extracts could modulate humoral immunity in ducks. The mechanism by which honeycomb extract enhanced immune functions is likely to depend on the synergistic action between the flavonoids, the phenolic acids in honeycomb extracts, and positively charged amino groups of chitosan, resulting in a reduction in bacterial growth (55).

Serum SOD activity was higher in laying ducks fed the honeycomb extract diet than in laying ducks fed the control diet in the current study. Some studies have shown that supplementation with flavonoids in poultry diets significantly enhanced serum antioxidant ability (4). Wang et al. (56) reported that supplementation with flavonoids in broiler chicken diets resulted in a significant increase in the level of reduced glutathione in the liver. Guo et al. (57) reported that flavonoids have antioxidant and free radical scavenging properties, which have been shown to prevent low-density lipoprotein peroxidation induced by copper ions. The mechanism by which honeycomb extract protect against reactive oxygen species is likely to be the inhibition of xanthine oxidase activity, which significantly prevents the formation of free radicals (58). However, the most important antioxidant mechanisms of honeycomb extracts are the repairability of the DNA damage caused by free radicals and the effect of scavenging reactive oxygen species in tissues, as well as the degradation properties of the signal cascade that causes lipid peroxidation (59, 60).

## 5. Conclusion

This study indicated that supplementation with 1.5 g/kg honeycomb extracts increased crude protein, improved fatty acid composition, enhanced contents of total amino acid and flavor amino acid and decreased contents of cholesterol and TMA in eggs. Moreover, the serum antioxidant and immune status was also improved by supplementation of honeycomb extracts. Therefore, honeycomb extracts may be used in the feed of laying ducks to improve fatty acid composition and amino acid content and reduce cholesterol and TMA in eggs, which is beneficial to health and egg flavor.

## Data availability statement

The original contributions presented in the study are included in the article/supplementary material, further inquiries can be directed to the corresponding author.

## Ethics statement

The animal studies were approved by the Animal Ethics Committee of the Institute of Animal Husbandry and Veterinary, Jiangxi Academy of Agricultural Science (2010-JXAAS-XM-01). The studies were conducted in accordance with the local legislation and institutional requirements. Written informed consent was obtained from the owners for the participation of their animals in this study.

## Author contributions

XC: Conceptualization, Investigation, Methodology, Supervision, Visualization, Writing – original draft. PX: Formal analysis, Methodology, Writing – original draft. WSo: Investigation, Software, Supervision, Visualization, Writing – original draft. QS: Software, Writing – original draft. ZZ: Funding acquisition, Project administration, Resources, Validation, Visualization, Writing – review & editing. JH: Validation, Writing – review & editing. JC: Writing – review & editing. CX: Writing – review & editing, Formal analysis. WSu: Writing – review & editing. GA: Data curation, Writing – review & editing. QW: Conceptualization, Funding acquisition, Project administration, Supervision, Visualization, Writing – review & editing.

## References

[1] Tian Y Zhang R Li G Zeng T Chen L Xu W. Microbial fermented feed affects flavor amino acids and yolk trimethylamine of duck eggs via cecal microbiota–yolk metabolites crosstalk. Food Chem. (2024) 430:137008. doi: 10.1016/j.foodchem.2023.137008, PMID: 37586289

[2] Jiang W Wu P Tang R Liu Y Kuang S Jiang J. Nutritive values, flavor amino acids, healthcare fatty acids and flesh quality improved by manganese referring to up-regulating the antioxidant capacity and signaling molecules TOR and Nrf2 in the muscle of fish. Food Res Int. (2016) 89:670–8. doi: 10.1016/j.foodres.2016.09.020, PMID: 28460965

[3] Göçmen R Gülşah K Cufadar Y. The use of different fat sources on performance, egg quality and egg yolk fatty acids content in laying quails. Turk J Agri Food Sci Tech. (2021) 9:1413–8. doi: 10.24925/turjaf.v9i8.1413-1418.4243

[4] Farahat MH Abdallah FM Ali HA. Effect of dietary supplementation of grape seed extract on the growth performance, lipid profile, antioxidant status and immune response of broiler chickens. Animal. (2016) 11:771–7. doi: 10.1017/S1751731116002251, PMID: 27804907

[5] Saleh AA El-Awady A Amber K Eid YZ Alzawqari MH Selim S. Effects of sunflower meal supplementation as a complementary protein source in the laying hen’s diet on productive performance, egg quality, and nutrient digestibility. Sustainability. (2021) 13:3557. doi: 10.3390/su13063557

[6] Tadesse D Retta N Girma M Ndiwa N Dessie T Hanotte O. Yolk fatty acid content, lipid health indices, and oxidative stability in eggs of slow-growing Sasso chickens fed on flaxseed supplemented with plant polyphenol extracts. Foods. (2023) 12:1819. doi: 10.3390/foods12091819, PMID: 37174357 PMC10178081

[7] Song B. Functions of cholesterol in development and atherosclerotic cardiovascular disease In: . The fourth symposium on metabolic biology of Chinese biophysical society. China: Guilin (2021). 43.

[8] Hobson-Frohock A Land DG Griffiths NM Curtis RF. Egg taints: association with trimethylamine. Nature. (1973) 243:304–5. doi: 10.1038/243304a0 4743217

[9] Li X Yuan G Chen X Guo Y Yang N Pi J. Fishy odor and TMA content levels in duck egg yolks. J Food Sci. (2018) 83:39–45. doi: 10.1111/1750-3841.13977, PMID: 29210463

[10] Sonnenburg J Bäckhed F. Diet–microbiota interactions as moderators of human metabolism. Nature. (2016) 535:56–64. doi: 10.1038/nature18846, PMID: 27383980 PMC5991619

[11] Dantzer R Mormede P. Stress in farm animals: a need for reevaluation. J Anim Sci. (1983) 57:6–18. doi: 10.2527/jas1983.5716, PMID: 6350254

[12] Zhu L Liao R Wu N Zhu G Yang C. Heat stress mediates changes in fecal microbiome and functional pathways of laying hens. Appl Microbiol Biotechnol. (2018) 103:461–72. doi: 10.1007/s00253-018-9465-8, PMID: 30368579

[13] Surai PF Fisinin VI. Vitagenes in poultry production: part 1. Technological and environmental stresses. World Poult Sci J. (2016) 72:721–34. doi: 10.1017/S0043933916000714

[14] Arthur J In: Hester PY, editor. Chapter 3 – duck eggs, in egg innovations and strategies for improvements. San Diego: Academic Press (2017). 23–32.

[15] Mori H Takaya M Nishimura K Goto T. Breed and feed affect amino acid contents of egg yolk and eggshell color in chickens. Poultry Sci. (2020) 99:172–8. doi: 10.3382/ps/pez557, PMID: 32416798 PMC7587729

[16] Gómez-Caravaca AM Gómez-Romero M Arráez-Román D. Advances in the analysis of phenolic compounds in products derived from bees. J Pharm Biomed Anal. (2006) 41:1220–34. doi: 10.1016/j.jpba.2006.03.002, PMID: 16621403

[17] Kang DH Kim MY. Comparative phenolic composition and antioxidant properties of honey and honeycomb extracts. J Life Sci. (2015) 25:1169–75. doi: 10.5352/jls.2015.25.10.1169

[18] Yang XZ Liu GY Liang JP Yang Q Guo YJ Tian MH. Extraction technology and antioxidant activity of flavonoids, polyphenols, polysaccharides in different parts of *Vespa velutina auraria* honeycomb. Southwest China J Agri Sci. (2020) 33:1679–84. doi: 10.16213/j.cnki.scjas.2020.8.012

[19] Yangi B Ustuner MC Dincer M Ozbayer C Tekin N Ustuner D. Propolis protects endotoxin induced acute lung and liver inflammation through attenuating inflammatory responses and oxidative stress. J Med Food. (2018) 21:1096–105. doi: 10.1089/jmf.2017.0151, PMID: 29719160

[20] Rzepecka-Stojko A Stojko J Kurek-Górecka A Górecki M Kabała-Dzik A Kubina R. Polyphenols from bee pollen: structure, absorption, metabolism and biological activity. Molecules. (2015) 20:21732–49. doi: 10.3390/molecules201219800 26690100 PMC6332396

[21] Xu XQ Yu L Liu SW. Protective and antioxidant properties of wasp (*Vespa magnifica*) honeycomb extract: a potential inhibitor against acidified ethanol-induced gastric lesions. J Southern Med Univ. (2011) 31:1107–10. doi: 10.1007/s10570-010-9464-0, PMID: 21764674

[22] Babaei S Rahimi S Torshizi MAK Tahmasebi G Miran SNK. Effects of propolis, royal jelly, honey and bee pollen on growth performance and immune system of Japanese quails. Vet Res Forum. (2016) 7:13–20. doi: 10.1016/b978-1-4377-2333-5.00068-7, PMID: 27226882 PMC4867032

[23] de Carvalho FMA Schneider JK de Jesus CVF de Andrade LN Amaral RG David JM. Brazilian red propolis: extracts production, physicochemical characterization, and cytotoxicity profile for antitumor activity. Biomol Ther. (2020) 10:726. doi: 10.3390/biom10050726, PMID: 32384801 PMC7277404

[24] Abass AO Kamel NN Khalifa WH Gouda GF El-Manylawi MAF Mehaisen GMK. Propolis supplementation attenuates the negative effects of oxidative stress induced by paraquat injection on productive performance and immune function in Turkey poults. Poultry Sci. (2017) 96:4419–29. doi: 10.3382/ps/pex248, PMID: 29053856 PMC7107162

[25] Zhu L Li J Wei C Luo T Deng Z Fan Y. A polysaccharide from *Fagopyrum esculentum* Moench bee pollen alleviates microbiota dysbiosis to improve intestinal barrier function in antibiotic-treated mice. Food Funct. (2020) 11:10519–33. doi: 10.1039/D0FO01948H, PMID: 33179663

[26] Bonamigo T Campos JF Oliveira AS Torquato HFV Balestieri JBP Cardoso CAL. Antioxidant and cytotoxic activity of propolis of *Plebeia droryana* and *Apis mellifera* (Hymenoptera, Apidae) from the Brazilian Cerrado biome. PLoS One. (2017) 12:e0183983. doi: 10.1371/journal.pone.0183983, PMID: 28898258 PMC5595300

[27] Song WJ Song QL Chen XL Liu GH Zou ZH Tan J. Effects of honeycomb extract on the growth performance, carcass traits, immunity, antioxidant function and intestinal microorganisms of yellow bantam broilers. Poultry Sci. (2022) 101:101811. doi: 10.1016/j.psj.2022.101811 35709681 PMC9207294

[28] Association of Official Analytical Chemists. Official methods of analysis. 17th ed. Arlington, VA, USA: AOAC (2000).

[29] Zhang R Li L Liu S Chen R Rao P. An improved method of cholesterol determination in egg yolk by HPLC. J Food Biochem. (1999) 23:351–61. doi: 10.1111/j.1745-4514.1999.tb00025.x 11326993

[30] Cullere M Tasoniero G Giaccone V Acuti G Marangon A Dalle ZA. Black soldier fly as dietary protein source for broiler quails: meat proximate composition, fatty acid and amino acid profile, oxidative status and sensory traits. Animal. (2018) 12:640–7. doi: 10.1017/s1751731117001860, PMID: 28735587

[31] Zhang AQ Mitchell SC Smith RL. Dietary precursors of trimethylamine in man: a pilot study. Food Chem Toxicol. (1999) 37:515–20. doi: 10.1016/S0278-6915(99)00028-9 10456680

[32] Iskender H Yenice G Dokumacioglu E Kaynar O Hayirli A Kaya A. Comparison of the effects of dietary supplementation of flavonoids on laying hen performance, egg quality and egg nutrient profile. Brit Poultry Sci. (2017) 58:550–6. doi: 10.1080/00071668.2017.1349297, PMID: 28682120

[33] Ting S Yeh HS Lien TF. Effects of supplemental levels of hesperetin and naringenin on egg quality, serum traits and antioxidant activity of laying hens. Anim Feed Sci Tech. (2011) 163:59–66. doi: 10.1016/j.anifeedsci.2010.10.001

[34] Surai PF. Polyphenol compounds in the chicken/animal diet: from the past to the future. J Anim Physiol Anim Nutr. (2014) 98:19–31. doi: 10.1111/jpn.12070 23527581

[35] Zhou Y Zhao R Li LU Chen W Chen J. Effect of daidzein on egg laying performance and hormone levels in serum of Shaoxing ducks during the late stage of egg production cycle. J Nanjing Agr Univ. (2002) 46:175–81. doi: 10.1080/00071660500064808

[36] Lien TF Yeh HS Su WT. Effect of adding extracted hesperetin, naringenin and pectin on egg cholesterol, serum traits and antioxidant activity in laying hens. Arch Anim Nutr. (2008) 62:33–43. doi: 10.1080/17450390701780318, PMID: 18341078

[37] Goliomytis M Orfanou H Petrou E Charismiadou MA Simitzis PE Deligeorgis SG. Effect of hesperidin dietary supplementation on hen performance, egg quality and yolk oxidative stability. Br Poult Sci. (2014) 55:98–104. doi: 10.1080/00071668.2013.870328, PMID: 24397432

[38] Chambers JR Zaheer K Akhtar H Abdel-Aal EM In: Hester PY, editor. Chapter 1 – chicken eggs, in egg innovations and strategies for improvements. San Diego: Academic Press (2017). 3–11.

[39] Ngo Njembe MT Dormal E Gardin C Mignolet E Debier C Larondelle Y. Effect of the dietary combination of flaxseed and Ricinodendron heudelotii or *Punica granatum* seed oil on the fatty acid profile of eggs. Food Chem. (2021) 344:128668. doi: 10.1016/j.foodchem.2020.128668, PMID: 33267981

[40] Yaplito-Lee J Pai G Hardikar W Hong KM Pitt J Marum J. Successful treatment of lathosterolosis: a rare defect in cholesterol biosynthesis—a case report and review of literature. JIMD Rep. (2020) 56:14–9. doi: 10.1002/jmd2.12158, PMID: 33204591 PMC7653246

[41] Jeong SM Choi S Kim K Kim SM Lee G Park SY. Effect of change in total cholesterol levels on cardiovascular disease among young adults. J Am Heart Assoc. (2018) 7:e008819. doi: 10.1161/JAHA.118.008819, PMID: 29899019 PMC6220545

[42] Kim HK Jeong TS Lee MK Yong BP Choi MS. Lipid-lowering efficacy of hesperetin metabolites in high-cholesterol fed rats. Clin Chim Acta. (2003) 327:129–37. doi: 10.1016/S0009-8981(02)00344-3, PMID: 12482628

[43] Liu Y Li Y Liu HN Suo YL Hu LL Feng XA. Effect of quercetin on performance and egg quality during the late laying period of hens. Br Poult Sci. (2013) 54:510–4. doi: 10.1080/00071668.2013.799758, PMID: 23906219

[44] You Y Han C Tabassum CM Li L Yao J Wang S. Effect of quercetin on egg quality and components in laying hens of different weeks. J Northeast Agr Univ. (2015) 22:23–32. doi: 10.1016/S1006-8104(16)30015-0

[45] Lee MK Moon SS Lee SE Bok SH Jeong TS Park YB. Naringenin 7-O-cetyl ether as inhibitor of HMG-CoA reductase and modulator of plasma and hepatic lipids in high cholesterol-fed rats. Bioorg Med Chem. (2003) 11:393–8. doi: 10.1016/S0968-0896(02)00441-8, PMID: 12517434

[46] Pieczynska MD Yang Y Petrykowski S Horbanczuk OK Atanasov AG Horbanczuk JO. Gut microbiota and its metabolites in atherosclerosis development. Molecules. (2020) 25:594. doi: 10.3390/molecules25030594, PMID: 32013236 PMC7037843

[47] Huang S Rutkowsky JM Snodgrass RG Ono-Moore KD Schneider DA Newman JW. Saturated fatty acids activate TLR-mediated proinflammatory signaling pathways. J Lipid Res. (2012) 53:2002–13. doi: 10.1194/jlr.D029546, PMID: 22766885 PMC3413240

[48] Holland WL Bikman BT Wang LP Guan Y Sargent KM Sarada B. Lipid-induced insulin resistance mediated by the proinflammatory receptor TLR4 requires saturated fatty acid–induced ceramide biosynthesis in mice. J Clin Invest. (2011) 121:1858–70. doi: 10.1172/JCI43378, PMID: 21490391 PMC3083776

[49] Garg P Pejaver RK Sukhija M Ahuja A. Role of DHA, ARA, & phospholipids in brain development: an Indian perspective. Clin Epidemiol Glob Health. (2017) 5:155–62. doi: 10.1016/j.cegh.2017.09.003

[50] Habermann N Schön A Lund EK Glei M. Fish fatty acids alter markers of apoptosis in colorectal adenoma and adenocarcinoma cell lines but fish consumption has no impact on apoptosis-induction ex vivo. Apoptosis. (2010) 15:621–30. doi: 10.1007/s10495-010-0459-y, PMID: 20107900

[51] Hodson L Skeaff CM Chisholm W. The effect of replacing dietary saturated fat with polyunsaturated or monounsaturated fat on plasma lipids in free-living young adults. Eur J Clin Nutr. (2001) 55:908–15. doi: 10.1038/sj.ejcn.1601234 11593354

[52] Tian XZ Li JX Luo QY Wang X Xiao MM Zhou D. Effect of supplementation with selenium-yeast on muscle antioxidant activity, meat quality, fatty acids and amino acids in goats. Front Vet Sci. (2022) 8:813672. doi: 10.3389/fvets.2021.813672, PMID: 35146016 PMC8821878

[53] Li J Zhou D Li H Luo Q Wang X Qin J. Effect of purple corn extract on performance, antioxidant activity, egg quality, egg amino acid, and fatty acid profiles of laying hen. Front Vet Sci. (2023) 9:1083842. doi: 10.3389/fvets.2022.1083842, PMID: 36686183 PMC9853176

[54] Khodabakhshi D Eskandarinia A Kefayat A Rafienia M Navid S Karbasi S. In vitro and in vivo performance of a propolis-coated polyurethane wound dressing with high porosity and antibacterial efficacy. Colloids Surf B Biointerfaces. (2019) 178:177–84. doi: 10.1016/j.colsurfb.2019.03.010, PMID: 30856587

[55] Pobiega K Kraniewska K Gniewosz M. Application of propolis in antimicrobial and antioxidative protection of food quality – a review. Trends Food Sci Tech. (2019) 83:53–62. doi: 10.1016/j.tifs.2018.11.007

[56] Wang ML Suo X Gu JH Zhang WW Fang Q Wang X. Influence of grape seed proanthocyanidin extract in broiler chickens: effect on chicken coccidiosis and antioxidant status. Poultry Sci. (2008) 87:2273–80. doi: 10.3382/ps.2008-00077, PMID: 18931178

[57] Guo XF Yue YD Tang F Wang J Yao X. Detection of antioxidative capacity of bamboo leaf extract by scavenging superoxide anion free radical. Spectrosc Spect Anal. (2008) 28:1823–6. doi: 10.3964/j.issn.1000-0593.2008.08.031, PMID: 18975812

[58] Di Carlo G Mascolo N Izzo AA Capasso F. Flavonoids: old and new aspects of a class of natural therapeutic drugs. J Life Sci. (1999) 65:337–53. doi: 10.1016/S0024-3205(99)00120-4, PMID: 10421421

[59] Isla MI Nieva Moreno MI Sampietro AR Vattuone MA. Antioxidant activity of Argentine propolis extracts. J Ethnopharmacol. (2001) 76:165–70. doi: 10.1016/S0378-8741(01)00231-8, PMID: 11390131

[60] Kumazawa S Hamasaka T Nakayama T. Antioxidant activity of propolis of various geographic origins. Food Chem. (2004) 84:329–39. doi: 10.1016/s0308-8146(03)00216-4

